# Primary Mesenteric Follicular Lymphoma Associated with Mesenteric Migration of Intrauterine Device

**DOI:** 10.4274/Tjh.2012.0207

**Published:** 2013-12-05

**Authors:** Xue-Feng Sun, Jun Feng, Wei Liu

**Affiliations:** 1 Peking Union Medical College Hospital, Department of Respiratory Medicine, Beijing, China; 2 Peking Union Medical College Hospital, Department of Hematology, Beijing, China; 3 Peking Union Medical College Hospital, Department of General Surgery, Beijing, China

**Keywords:** Mesenteric follicular lymphoma, Intrauterine device

A 39-year-old woman received an intrauterine device (IUD) placement 6 years ago, and lower abdominal pain occurred intermittently 1 year after the placement. Every attack lasted for a few days and was relieved spontaneously. Pain occurred again 2 days before admission, with nausea, vomiting, and constipation. Physical examination revealed a mass in the left lower abdomen with localized tenderness. Abdominal computed tomography showed that a mass, 5.9 × 4.0 cm in size, was adjacent to the aorta abdominalis and below the umbilicus. A 2-cm circinate foreign body was observed close to the mass (Figure 1). Exploratory laparotomy showed that a metallic IUD was on the mesenterium, while the uterine was intact, and the mesenterium, 130 cm distal to the Treitz ligament until 40 cm proximal to the ileocecum, was occupied by purple nodules, which coalesced into a large mass (Figure 2). Diseased mesenterium and relevant intact small bowel were resected successfully. Postoperative pathologic study showed abnormal crowding of follicles and many large centroblasts with nucleoli adjacent to the nuclear membrane with admixed cleaved cells, in accordance with Grade III follicular lymphoma (Figure 3). Immunohistochemistry results were positive for CD20, CD21, mum-1, Bcl-2, and Bcl-6 and weakly positive for CD10, with a Ki-67 index of 60%. Further bone marrow smear and biopsy had negative results. She was treated with 6 cycles of combined chemotherapy after surgery, and no relapse was observed at the 1-year follow-up. Informed consent was obtained. 

IUDs become embedded in the uterine wall or even perforated into the peritoneal cavity only in very rare cases. It has been reported that perforation of the uterus occurs in 0.87 of 1000 insertions [1]. Follicular lymphoma is an indolent lymphoma and accounts for about 10% of non-Hodgkin lymphomas in China. Although the presence of primary follicular lymphoma of the gastrointestinal tract is relatively commonly seen, primary mesenteric follicular lymphoma is extremely rare. 

It is usually considered that IUDs are unrelated to malignancy, and might even act as a protective cofactor in cervical carcinogenesis [2], but there are no studies about the safety of migrated IUDs. In this case, mesenteric follicular lymphoma followed IUD placement and migration, and they were adjacent in space, which brought about the postulation that there was some unusual association between the formation of follicular lymphoma and the migrated IUD. This might be explained by the fact that the pathogenesis of lymphoma is associated with inflammation [3]. 

## CONFLICT OF INTEREST STATEMENT

The authors declare that they have no conflicts of interest that could be perceived as having influenced the impartiality of the materials presented. 

**Funding**

The present study received no grant from a funding agency in the public, commercial, or for-profit sectors. 

## Figures and Tables

**Figure 1:**
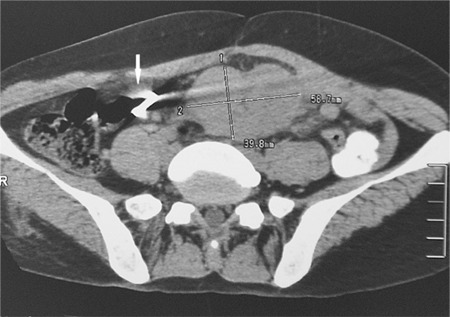
Abdominal CT reveals a mass of 5.9 x 4.0 cm in size adjacent to the aorta abdominalis under the umbilicus, to the right of which a dissociative circinate foreign body of 2 cm in diameter is observed (arrow).

**Figure 2:**
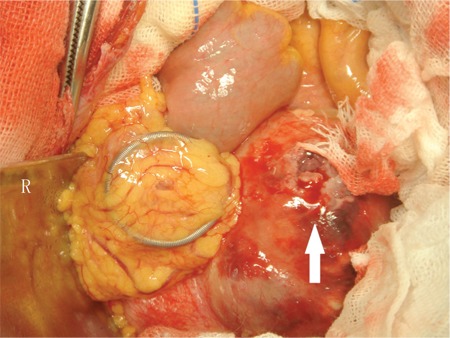
During laparotomy, a metallic IUD was noticed on the mesenterium. The mesenterium to the left of the IUD is occupied by purple nodules coalescing into a large mass. Some nodules were ruptured, and brown mucus was seen flowing out.

**Figure 3:**
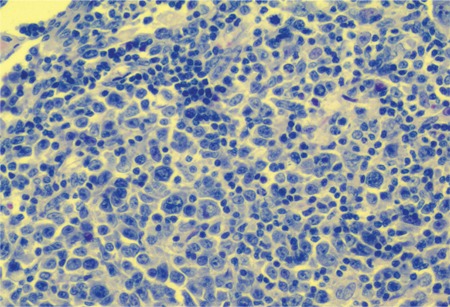
Ostoperative pathologic study showed abnormal crowding of follicles and many large centroblasts with nucleoli adjacent to the nuclear membrane with admixed cleaved cells, in accordance with Grade III follicular lymphoma (hematoxylin and eosin, 100x).
